# Cerebral Embolism Caused by Pulmonary Vein Stump Compression Due to Postoperative Exacerbation of Hiatal Hernia After Left Lower Lobectomy: A Case Report

**DOI:** 10.7759/cureus.104210

**Published:** 2026-02-24

**Authors:** Kenji Shono, Yu Sato, Eiji Shikata, Shunji Matsubara, Norihito Shirakawa

**Affiliations:** 1 Neurosurgery, Takamatsu Municipal Hospital, Takamatsu, JPN

**Keywords:** cerebral infarction, hiatal hernia, pulmonary vein thrombosis, thoracic surgery, thrombectomy

## Abstract

Cerebral infarction after lung cancer surgery is a critical complication typically associated with thrombus formation in the long pulmonary vein stump, particularly after left upper lobectomy (LUL). Conversely, the risk of thrombus formation after left lower lobectomy (LLL) is low because of the anatomically shorter stump. A man in his 70s underwent video-assisted thoracoscopic surgery (VATS) left lower lobectomy for lung cancer. On postoperative day 1, the patient developed sudden right hemiplegia, global aphasia, and left conjugate deviation. Magnetic resonance imaging (MRI) revealed an acute cerebral infarction due to left middle cerebral artery (M1) occlusion. As intravenous thrombolysis was contraindicated in the immediate postoperative period, emergent mechanical thrombectomy using a direct aspiration first-pass technique (ADAPT) was performed to achieve complete recanalization (TICI 3). ADAPT thrombectomy is a safe and effective preferred strategy for acute macrovascular occlusion that cannot be intravenously thrombolysis in the early postoperative period. Postoperative investigation revealed an acute exacerbation of the hiatal hernia, which physically compressed and obstructed the stump of the left inferior pulmonary vein. The retrieved thrombus was a mixed thrombus composed of erythrocytes and fibrin, consistent with the formation due to stasis in an obstructed space. LLL is an independent risk factor for postoperative hiatal hernia exacerbation. This case suggests that even an anatomically "short stump" can develop severe hemodynamic stasis leading to thrombosis if subjected to external mechanical compression. Clinicians must recognize that even after LLL, anatomical changes, such as hiatal hernia exacerbation, can introduce a risk of pulmonary vein thrombosis and subsequent cerebral embolism.

## Introduction

The incidence of cerebral infarction following lung cancer surgery is reported to be 0.2% to 1.1% [1]. Although rare, this is a devastating complication that can significantly impair patient prognosis. Primary mechanisms include blood stasis and thrombus formation within the pulmonary vein stump (PVS). Left upper lobectomy (LUL) is a known risk factor because the left superior PVS tends to remain anatomically long (median, approximately 1.71 cm), creating a cul-de-sac that favors thrombus formation and increases the risk of cerebral infarction compared with other lobectomies [2,3]. In contrast, the stump following left lower lobectomy (LLL) is typically short (median, approximately 0.54 cm), and the risk of stump thrombosis is considered low [2].

Large hiatal hernias are a rare cause of pulmonary vein thrombosis and systemic embolism due to prolapsed organs physically compressing the left atrium or pulmonary veins, causing hemodynamic disturbances [4,5]. Thoracic surgery, particularly LLL, carries a high risk of inducing or exacerbating hiatal hernias due to enlargement of the thoracic space and changes in pressure gradients [6]. However, reports directly linking hernia exacerbation to thrombus formation in the inferior PVS and the subsequent cerebral infarction are rare.

We report a case of acute cerebral infarction after left lower lobectomy, in which an acute exacerbation of a hiatal hernia mechanically compressed and occluded the stump of the left inferior pulmonary vein, which is generally considered to be at low risk of thrombosis. We discuss the pathogenic mechanisms and the favorable outcome after prompt mechanical thrombectomy using a direct aspiration first-pass technique (ADAPT).

## Case presentation

Patient presentation

A 70-year-old man who had undergone video-assisted thoracoscopic surgery (VATS) for left lower lobectomy for lung cancer at our institution exhibited a sudden disturbance of consciousness and right-sided weakness immediately after returning to his room from walking to the restroom on the evening of postoperative day 1. The surgery was completed without complications, and the immediate postoperative course was uneventful. Upon examination, the patient’s Glasgow Coma Scale (GCS) score was E3V1M5. Vital signs showed a blood pressure of 150/90 mmHg and a heart rate of 80 bpm (sinus rhythm). Neurological examination revealed left conjugate deviation, global aphasia, and right hemiplegia (Manual Muscle Test, 1/5). The National Institutes of Health Stroke Scale (NIHSS) score was 17.

Imaging and treatment course

**Figure 1:**
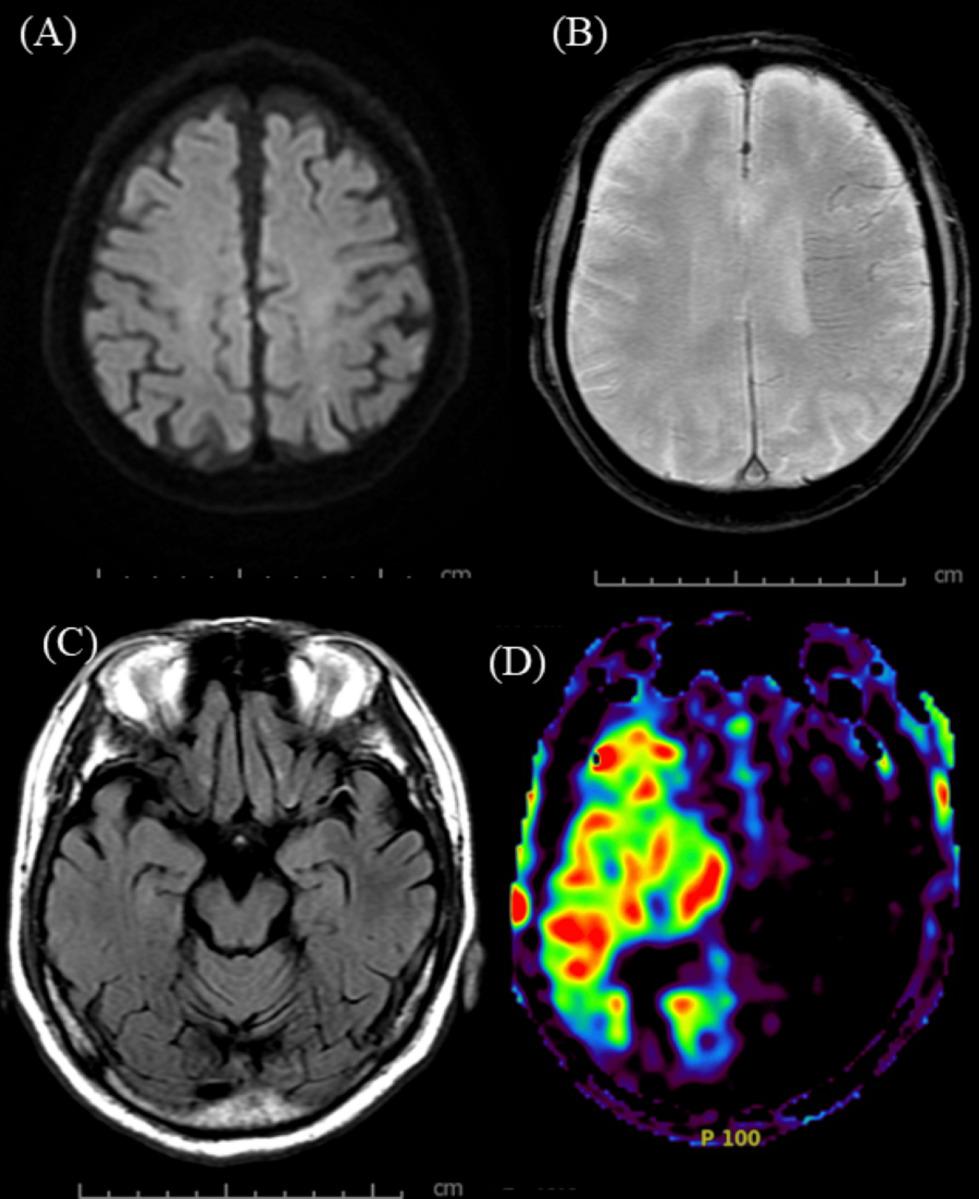
Preoperative magnetic resonance imaging (MRI) findings. (A) Diffusion-weighted imaging (DWI) reveals faint hyperintensity in the left parietal lobe. (B) T2*-weighted imaging demonstrates the brush sign along the periventricular medullary veins in the left hemisphere. (C) Fluid-attenuated inversion recovery (FLAIR) imaging shows linear hyperintensity (intra-arterial sign) within the left middle cerebral artery (MCA). (D) Arterial spin labeling (ASL) indicates extensive hypoperfusion in the left cerebral hemisphere.

An emergency head magnetic resonance imaging (MRI) was performed, and diffusion-weighted imaging (DWI) showed only a faint hyperintensity in the left parietal lobe due to the hyperacute phase. However, fluid attenuated inversion recovery (FLAIR) imaging revealed an intra-arterial sign [7] in the left middle cerebral artery (MCA) territory (Figure 1).

**Figure 2:**
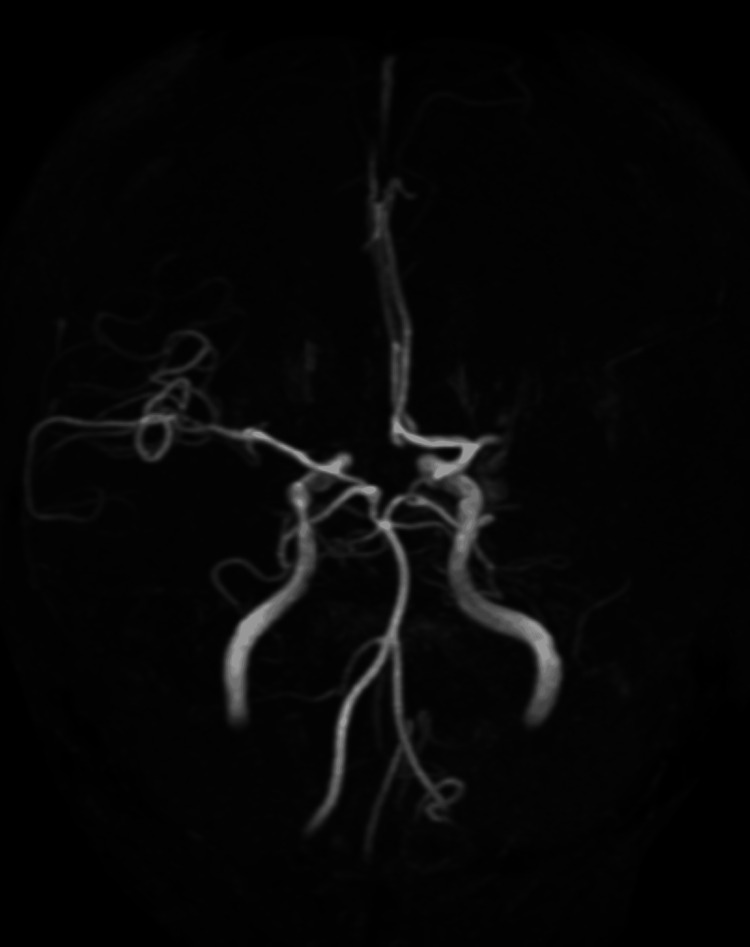
Preoperative magnetic resonance angiography (MRA). MRA confirms occlusion of the M1 segment of the left middle cerebral artery (MCA).

Additionally, T2*-weighted imaging showed a brush sign [8] in the deep white matter of the left cerebral hemisphere, suggesting extensive ischemia. Magnetic resonance angiography (MRA) confirmed signal loss in the proximal left MCA (M1 segment) and decreased arterial spin labeling (ASL) signal in the corresponding region (Figure 2).

**Figure 3:**
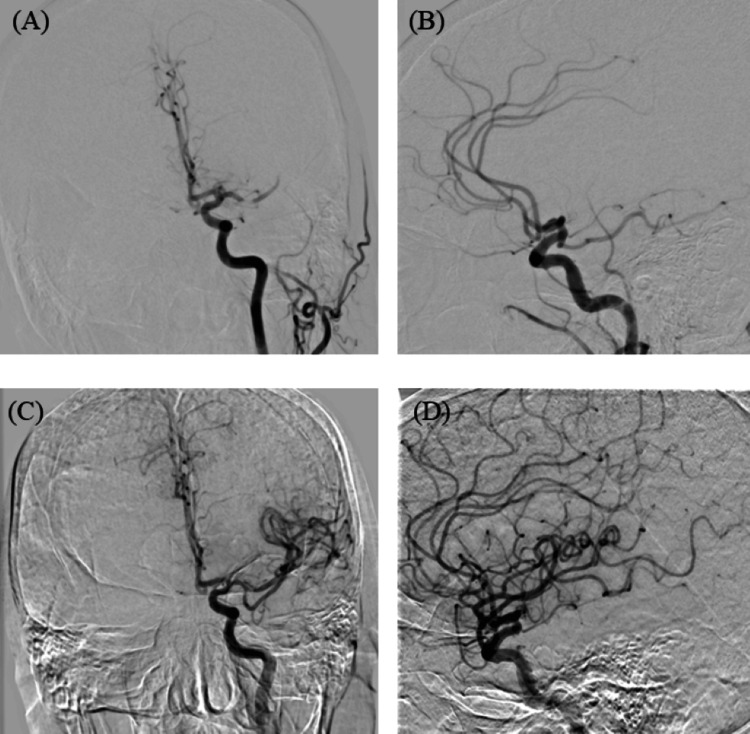
Digital subtraction angiography (DSA). (A) Pre-thrombectomy angiography (antero-posterior view) showing occlusion of the left middle cerebral artery. (B) Pre-thrombectomy angiography (lateral view). (C) Post-thrombectomy angiography (antero-posterior view) demonstrating complete recanalization (thrombolysis in cerebral infarction or TICI grade 3). (D) Post-thrombectomy angiography (lateral view).

The patient was diagnosed with an acute cerebral infarction due to left M1 occlusion. Since the patient underwent lung resection within 24 h, intravenous tissue plasminogen activator (t-PA) therapy was contraindicated because of the risk of hemorrhagic complications. The patient was immediately transferred to the endovascular therapy department. Groin puncture was performed 112 min after onset. Digital subtraction angiography confirmed the left M1 occlusion. We performed ADAPT using a large-bore aspiration catheter as the first-line strategy to achieve thrombectomy as quickly as possible. Complete recanalization (TICI 3) was achieved with a single pass (31 min from puncture to recanalization) (Figure 3).

Pathological findings

**Figure 4:**
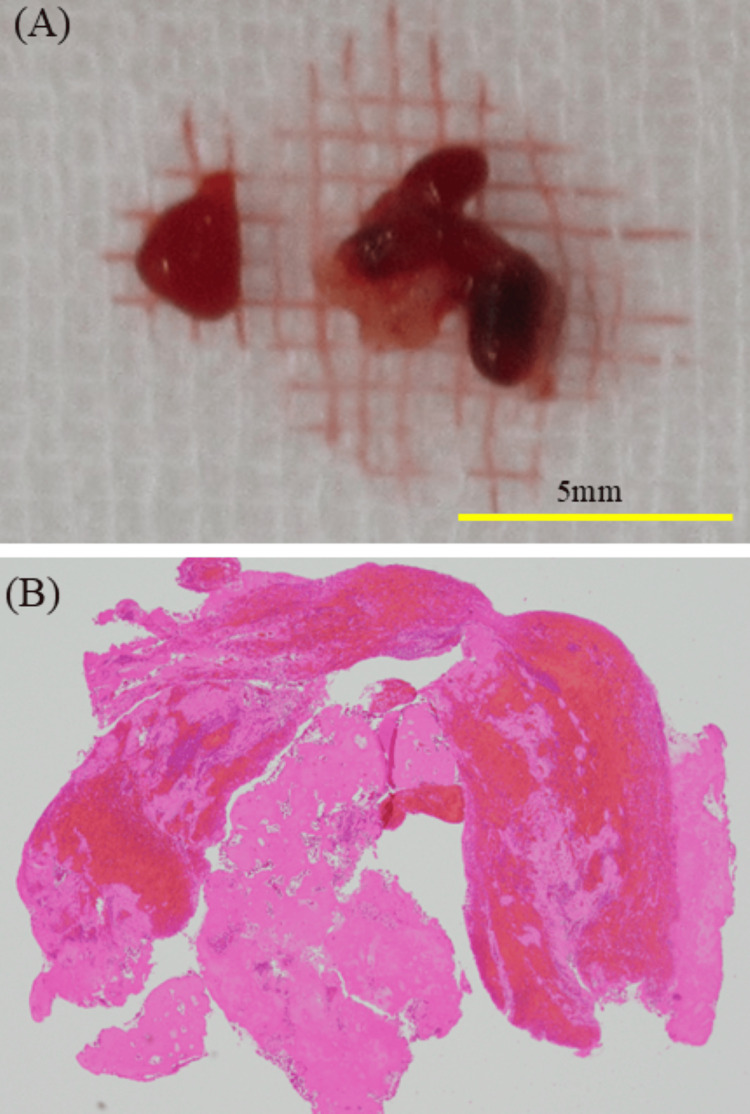
Macroscopic and pathological findings of the thrombus. (A) Macroscopic view of the retrieved thrombus. The yellow scale bar indicates 5 mm. (B) Histopathological examination (hematoxylin and eosin or H&E stain, original magnification ×10) reveals a mixed thrombus composed of erythrocytes and fibrin.

Histopathological examination of the retrieved red thrombus revealed a mixed thrombus containing erythrocytes, leukocytes, and fibrin (Figure 4). No atherosclerotic plaques were observed. These findings are consistent with a coagulation thrombus formed under stasis conditions.

Postoperative course and etiology

**Figure 5:**
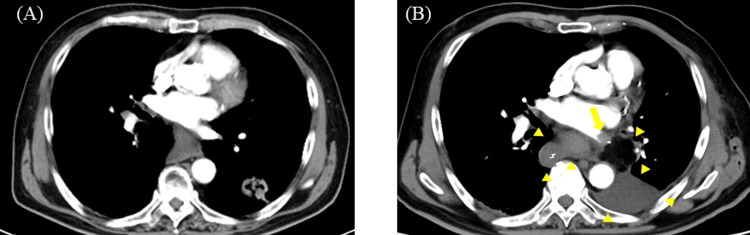
Contrast-enhanced chest computed tomography (CT). (A) Preoperative CT showing a mild hiatal hernia. (B) Post-thrombectomy CT. The left inferior pulmonary vein is compressed by an exacerbated hiatal hernia (arrowheads), and a filling defect is noted within the vein (arrow).

MRI performed immediately after thrombectomy showed scattered infarcts in the left basal ganglia and parietal lobe; however, the clinical symptoms improved dramatically, with resolution of hemiplegia and aphasia. Embolic source workup revealed no atrial fibrillation on continuous electrocardiogram monitoring, and carotid ultrasonography showed no significant stenosis. Postoperative contrast-enhanced chest computed tomography (CT) revealed marked exacerbation of the preexisting hiatal hernia. The stomach prolapsed into the thoracic cavity and compressed the left inferior pulmonary vein (LIPV) stump (Figure 5). A filling defect (residual thrombus) was identified within the LIPV stump. The LIPV stump length was 10 mm. Preoperative D-dimer was not measured. Based on these findings, the patient was diagnosed with cerebral embolism caused by pulmonary vein stump thrombosis (PVST) resulting from compression and obstruction by an exacerbated hiatal hernia following lung resection.

Outcome

**Figure 6:**
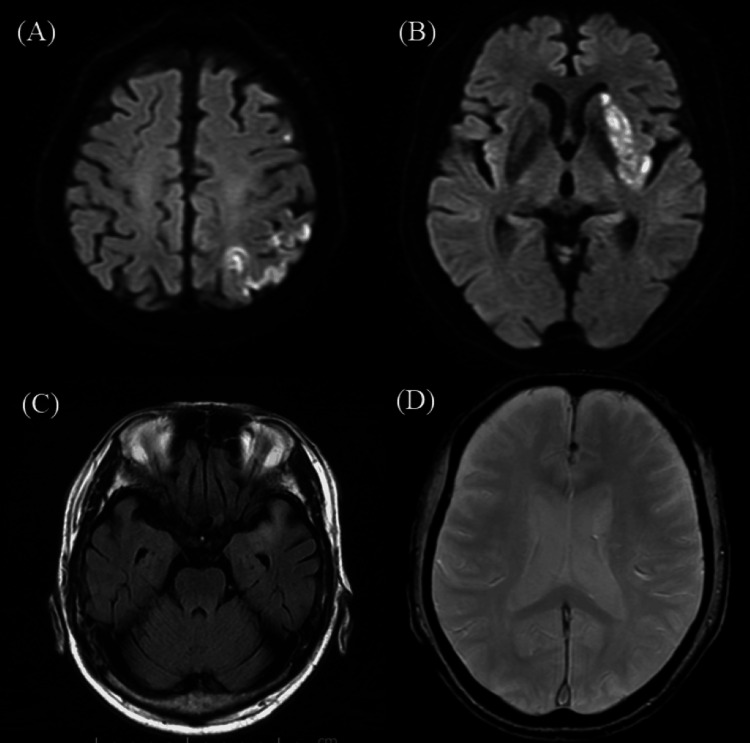
Post-procedural MRI findings. (A, B) Diffusion-weighted imaging (DWI) shows scattered hyperintensities in the left parietal lobe and basal ganglia. (C) Fluid-attenuated inversion recovery (FLAIR) imaging shows resolution of the intra-arterial signal in the middle cerebral artery (MCA). (D) T2*-weighted imaging shows the disappearance of the brush sign.

**Figure 7:**
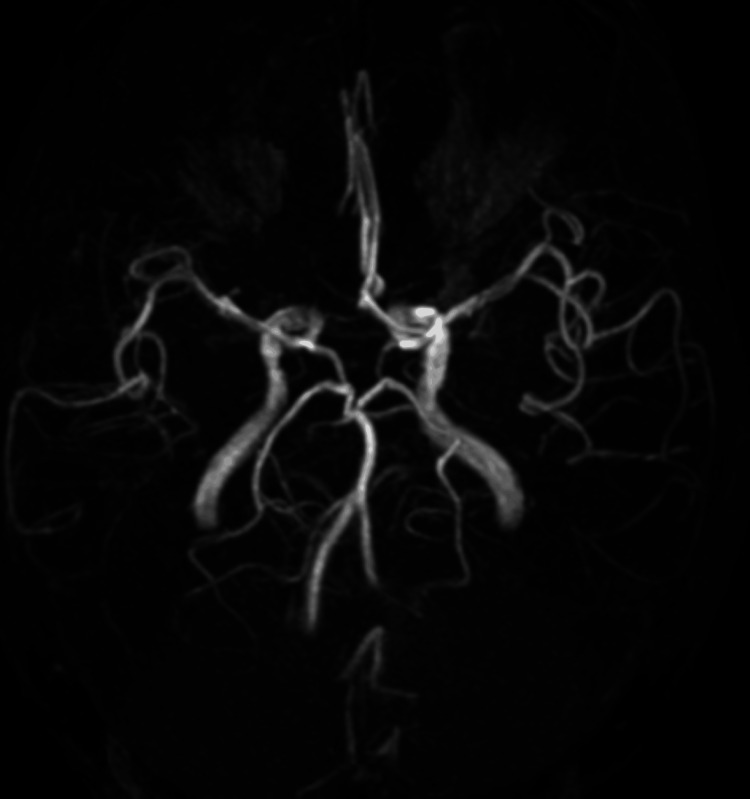
Post-procedural MRA. Magnetic resonance angiography (MRA) demonstrates complete patency of the left middle cerebral artery (MCA).

Anticoagulation therapy with heparin followed by warfarin was initiated. The D-dimer level was 1.3 µg/mL on the day after ADAPT thrombectomy, peaked at 5.1 µg/mL on postoperative day 4, and subsequently normalized approximately one month later. Postoperative MRI showed infarction in the left parietal lobe and basal ganglia; however, the intra-arterial and brush signs disappeared, and the M1 segment was patent on MRA (Figures 6, 7).

**Figure 8:**
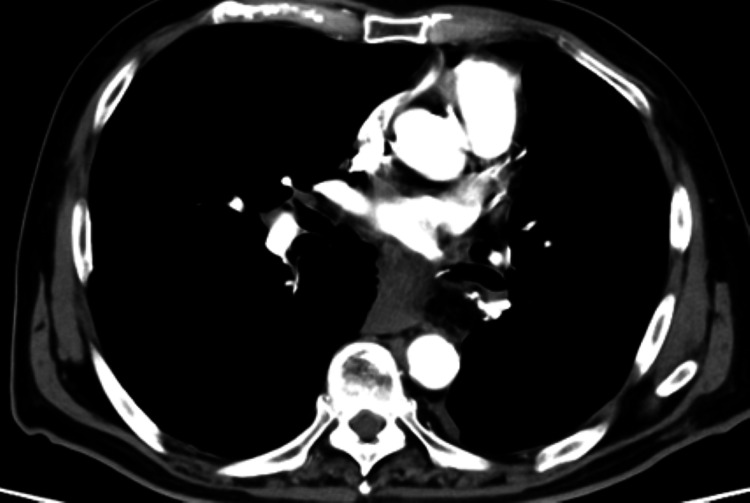
Follow-up contrast-enhanced computed tomography (CT). CT shows resolution of the thrombus in the left inferior pulmonary vein stump.

Follow-up contrast-enhanced CT confirmed the disappearance of the thrombus in the PVS (Figure 8).The patient was discharged on postoperative day 20, ambulatory, and independent (modified Rankin Scale 1), with no neurological sequelae. Informed consent was obtained from the patient for the publication of this case report.

## Discussion

This case represents an extremely rare instance of acute exacerbation of a hiatal hernia following lung cancer surgery, compressing the left inferior pulmonary vein stump, causing blood stasis, PVST, and subsequent cerebral embolism. The clinical significance of this case lies in demonstrating a novel pathogenic mechanism. Even after LLL, typically considered a low-risk procedure for thrombosis, the addition of physical compression by an exacerbated hernia can transform a "safe" short stump into a nidus for thrombus formation.

Mechanism of PVST and the specificity of this case

As reported by Ohtaka et al., PVST generally occurs after LUL [2]. This is because the left superior pulmonary vein stump is anatomically long (median: approximately 1.71 cm) and prone to turbulence and stasis. Conversely, the stump after LLL is short (median, approximately 0.54 cm), making thrombosis extremely rare [2]. An exception was reported by Usui et al., who described a case of cerebral infarction treated with thrombectomy after LLL [9]. However, they attributed this to an anatomically anomalous long stump (1.4 cm). This falls within the conventional theory that "a long stump constitutes the risk." In contrast, the residual stump in our case was 1.0 cm, shorter than that in the case of Usui et al., and is not typically considered a high-risk length. However, thrombosis was still observed [9]. We propose a "functional" etiology, namely, physical compression by the hiatal hernia.

Association between LLL and hiatal hernia

According to a large-scale study by Song et al., LLL is the strongest risk factor for new-onset or enlargement of hiatal hernias after thoracic surgery, with an incidence rate of 24.2% [6]. This was significantly higher than that of the LUL or right-sided resections. The increased space in the left thoracic cavity, diaphragmatic elevation, and manipulation of the inferior pulmonary ligament likely contributed to the manifestation of the hernia. In our case, marked exacerbation of the hernia was confirmed postoperatively. Saoraya et al. reported a nonsurgical case in which a large hiatal hernia directly compressed the pulmonary vein, causing stasis that led to pulmonary vein thrombosis and renal infarction [4]. Additionally, Naoum et al. demonstrated that large hernias can compress the left atrium and affect hemodynamics [5]. Our case combines two factors: the LLL procedure (prone to inducing hernias) and hernia-induced stump compression. This combination functionally transformed an anatomically low-risk 1.0 cm stump into an obstructed cul-de-sac, causing severe stasis and thrombosis. This finding serves as a warning against relying solely on "stump length" for risk assessments.

Thrombus characteristics and treatment

The retrieved thrombus was a red thrombus, which is typical of low-flow environments or obstructed spaces [10]. This differs from white thrombi caused by atherosclerotic plaque rupture and pathologically supports the hypothesis of rapid formation within the "stagnation" created by hernia compression. Systemic thrombolysis is contraindicated in the early postoperative period due to the risk of hemorrhage. Therefore, a mechanical thrombectomy is the preferred treatment option. Our department uses ADAPT as the first-line strategy to shorten the time from puncture to recanalization and reduce invasiveness. The COMPASS trial by Turk et al. demonstrated that ADAPT is noninferior to stent retrievers in terms of functional outcomes and significantly reduces procedural time [11]. In this case, involving a fresh thrombus formed early postoperatively, aspiration was particularly suitable, resulting in rapid TICI 3 recanalization and an excellent clinical outcome.

## Conclusions

We encountered a case of cerebral embolism caused by compression of the inferior pulmonary vein stump due to the acute exacerbation of a hiatal hernia following left lower lobectomy. Although LLL is generally considered to carry a low risk of thrombosis, clinicians should be aware that the exacerbation of a hiatal hernia can mechanically compress the vein, introducing a risk of thrombosis, even in short stumps. Furthermore, rapid mechanical thrombectomy using ADAPT is extremely effective for perioperative stroke where thrombolysis is contraindicated.
